# Achieving Boron–Carbon–Nitrogen Heterostructures by Collision Fusion of Carbon Nanotubes and Boron Nitride Nanotubes

**DOI:** 10.3390/molecules28114334

**Published:** 2023-05-25

**Authors:** Chao Zhang, Jiangwei Xu, Huaizhi Song, Kai Ren, Zhi Gen Yu, Yong-Wei Zhang

**Affiliations:** 1 School of Materials Science and Engineering, Anhui University of Science and Technology, Huainan 232001, China; xujw1022@163.com (J.X.); huaizhi.song@outlook.com (H.S.); 2 School of Mechanical and Electronic Engineering, Nanjing Forestry University, Nanjing 210042, China; kairen@njfu.edu.cn; 3 Institute of High Performance Computing (IHPC), Agency for Science, Technology and Research (A*STAR), 1 Fusionopolis Way, #16-16 Connexis, Singapore 138632, Singapore; yuzg@ihpc.a-star.edu.sg (Z.G.Y.); zhangyw@ihpc.a-star.edu.sg (Y.-W.Z.)

**Keywords:** heteronanotube, heteronanoribbon, collision dynamics, electronic properties, DFTB

## Abstract

Heterostructures may exhibit completely new physical properties that may be otherwise absent in their individual component materials. However, how to precisely grow or assemble desired complex heterostructures is still a significant challenge. In this work, the collision dynamics of a carbon nanotube and a boron nitride nanotube under different collision modes were investigated using the self-consistent-charge density-functional tight-binding molecular dynamics method. The energetic stability and electronic structures of the heterostructure after collision were calculated using the first-principles calculations. Five main collision outcomes are observed, that is, two nanotubes can (1) bounce back, (2) connect, (3) fuse into a defect-free BCN heteronanotube with a larger diameter, (4) form a heteronanoribbon of graphene and hexagonal boron nitride and (5) create serious damage after collision. It was found that both the BCN single-wall nanotube and the heteronanoribbon created by collision are the direct band-gap semiconductors with the band gaps of 0.808 eV and 0.544 eV, respectively. These results indicate that collision fusion is a viable method to create various complex heterostructures with new physical properties.

## 1. Introduction

In recent decades, research interest in nanomaterials’ synthesis has been growing rapidly, enabling the attainment of novel materials such as heterostructures [1,2,3,4,5,6]. These heterostructures often exhibit fascinating new physical phenomena and properties that are otherwise absent in their individual components [7,8,9,10,11]. Currently, it is still a significant challenge to precisely grow or assemble such heterostructures [12,13,14,15,16].

Carbon nanotubes (CNTs) [17], one of the most widely studied nanomaterials, can be regarded as one-dimensional nanomaterials with unique hollow structures formed by curling up graphene sheets. They can exhibit excellent electrical, thermal, mechanical and optical performances, and have been widely used in electronic devices, hydrogen storage, thermally conductive materials and composite materials [18,19,20,21]. Similar to CNTs, boron nitride nanotubes (BNNTs) can also be regarded as hollow tubes formed by rolling up hexagonal boron nitride (*h*-BN) sheets [22,23,24,25,26], which have a hexagonal layered structure similar to graphene. BNNTs are wide-band-gap semiconductors with high thermal conductivity, high oxidation resistance and excellent mechanical strength, and they have been widely used in the field of nanocomposites and hydrogen storage materials [27,28,29]. Note that BNNTs have a similar structure to CNTs and their difference is that only nitrogen and boron atoms are alternately replaced by carbon atoms. The lattice mismatch between BNNT and CNT is about 0.79%. This tiny lattice difference provides a possibility of designing heterostructural materials by combining these two nanostructures. At present, there are generally two ways to form heteronanotubes by combining BNNTs and CNTs. One way is to stack them together to form van der Waals co-axial bilayer heteronanotubes with CNT@BNNT, that is, single-walled BNNT-wrapped single-walled CNT (SWCNT) [30,31]. The CNT@BNNTs exhibit superior thermal stability, chemical inertness and mechanical robustness in comparison to CNTs [31]. Another method is to combine CNTs and BNNTs to form covalent heteronanotubes either along or perpendicular to the tube axis. The covalent heteronanotubes along the tube axis, which exhibit tunable electronic properties based on stoichiometry, were prepared by covalent doping of CNTs with BNNTs [31,32]. However, many complex covalent heteronanotubes, for example, perpendicular to the tube axis, have not been realized thus far. How to form various complex heteronanotubes remains largely unexplored, which motivates the present study. It is noted that doping into graphite or carbon nanotubes has been used to create BCN nanostructures, and the preparation method can be traced back to the work of Stephan et al. in 1994 [32]. Subsequently, B_x_C_y_N_z_, CB_x_N_y_, CB_x_ and CN_x_ nanotubes were also successfully prepared. A large number of studies [33,34] have demonstrated that the doping of boron and nitrogen into carbon nanotubes can be an effective way to adjust the band gap of the material [35]. Interestingly, Campbell et al. obtained direct experimental evidence of the collision fusion of two C_60_ molecules [36,37,38].

In this work, BCN nanotubes were achieved by colliding CNTs and BNNTs based on the computational simulation method. To demonstrate the viability of this method, the self-consistent-charge density-functional tight-binding (SCC-DFTB) molecular dynamics (MD) method was employed to simulate the collision process. The simulations clearly demonstrated the formation of BCN nanotubes, indicating the parallel collision method is viable for producing BCN nanotubes. Interestingly, heteronanoribbons of graphene and *h*-BN are also formed after collision. We further showed that the BCN nanotube and the heteronanoribbon formed by the collision are direct band-gap semiconductors with band gaps of 0.81 eV and 1.34 eV, respectively. The present study suggests that heteronanotubes and heteronanoribbons can be effectively achieved by the collision fusion of CNTs and BNNTs.

## 2. Collision Results

**Figure 1:**
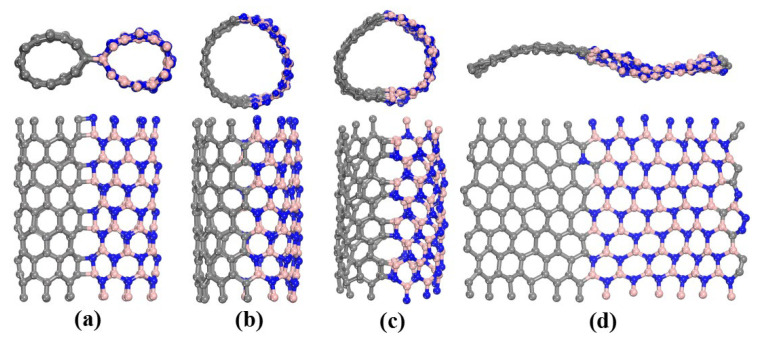
Typical structures formed by the collisions of CNTs and BNNTs. (**a**) Heterojunction, (**b**) defect-free (12,0) BCN nanotube, (**c**) BCN nanotube with four- and eight-membered ring defects, and (**d**) BCN nanoribbon.

There are five main collision outcomes that are observed in the simulations. I. The two nanotubes bounce off after collision and then move in opposite directions, labeled as B (see Video S1). Ⅱ. The two nanotubes are merged together in the form of *sp*^3^ hybridization after collision, labeled as C (see Figure 1a and Video S2). Ⅲ. The two nanotubes are fused into a perfect (12,0) BCN single-walled nanotube with a larger diameter without any defects, labeled as P (see Figure 1b and Video S3). Ⅳ. The two nanotubes are either fused to form a larger diameter BCN nanotube with defects, including a 4–8 ring defect, 5–7 pair defect [39], Stone–Wales (SW) defect [40], inverse Stone–Wales (ISW) defect [41], etc. (see Figure 1c), or form a quasi-one-dimensional BCN nanoribbon with six-membered rings at the junction and irregular defects at the edge (see Figure 1d and Video S4), labeled as D. Ⅴ. The two nanotubes are severely damaged after collision. With the increase in colliding energy, the structural damage becomes more and more serious, and the nanotubes may break up into single atoms, dimers, trimers or atomic chains, labeled as S.

**Figure 2:**
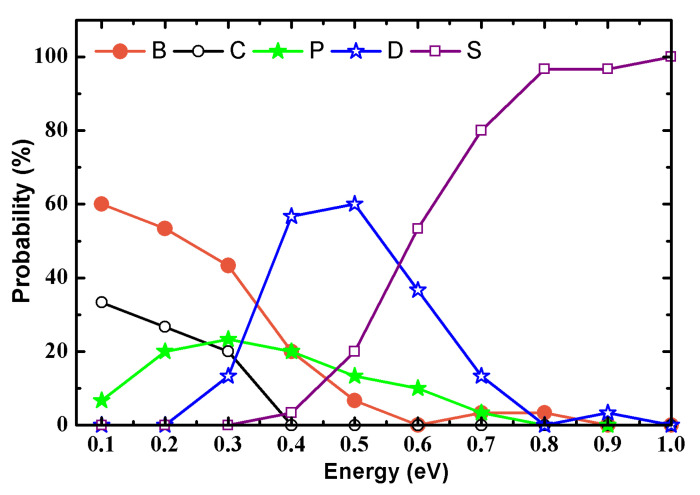
Probability of collision outcomes as a function of the initial collision energy. Solid circle, hollow circle, solid pentastar, hollow pentastar and hollow quadrangle represent the two nanotubes bouncing back (B) and connecting (C), fusing into BCN heteronanotubes without defects (P) and with defects (D) and colliding to create serious damage (S), respectively.

The probability of each collision outcome is shown in Figure 2. It can be seen that the probability of the two nanotubes being bounced back and forming a heterojunction is higher in the lower energy range (0.1~0.3 eV). In the medium-energy region (0.3~0.5 eV), the probability of the two tubes fusing to form nanotubes and nanoribbons is relatively high. In the high-energy region (0.5~1.0 eV), the probability of fused structures with severe damage is very high. The simulation results are summarized as shown in Table 1. It can be observed that the collision mode has a minor influence on the collision results. The connected heterojunctions mainly occur in the collision mode of AA, h-AH and AB, and the corresponding energy is in the range of 0.1 to 0.3 eV. The fusion of two nanotubes into a perfect defect-free BCN nanotube mainly occurs in three collision modes, i.e., h-AH, AH and AB. The fused BCN nanotubes and BCN nanoribbons with defective structures are found in each collision mode, and the corresponding energy is in the range of 0.3 to 0.7 eV.

## 3. Discussion

### 3.1. Collision Energetics

**Figure 3:**
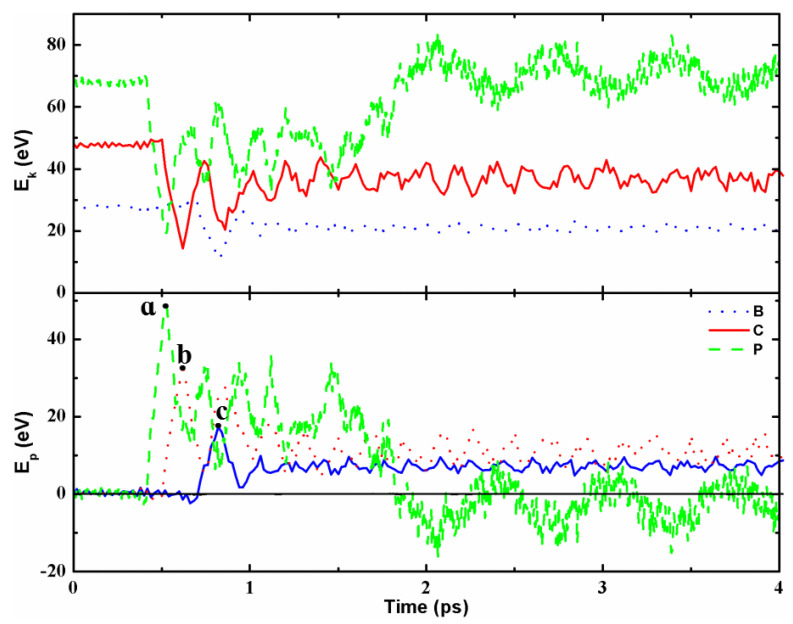
(Color online.) Time evolutions of the kinetic and potential energies of the two nanotubes bouncing back (B), connecting heterojunction (C) and fusing into a perfect BCN nanotube (P) after collision. The solid black line represents the total energy of the system, namely the sum of the kinetic and potential energies.

Figure 3 shows the time evolution of kinetic and potential energies of the two nanotubes bouncing back (B), connecting heterojunction (C) and fusing into a perfect BCN nanotube (P) after collision. It can be seen that all kinetic energy curves first drop sharply, then fluctuate up and down, and finally reach equilibrium. Because the total energy of the system remains constant, the evolution behavior of the corresponding potential energy is exactly opposite to that of the kinetic energy. In addition, for case B, the kinetic energy of the system after stabilization is lower than the initial kinetic energy, and the reduced kinetic energy has been transferred into the potential energy of the system, resulting in the increase in the potential energy, that is, the cylindrical tubular structure becomes an ellipsoidal cylinder after collision, as shown in Figure 3b and Video S1. For case C, the final kinetic energy is also lower than the initial kinetic energy. On the one hand, the reduced kinetic energy has been converted into the binding energy required for the formation of the junction between the two nanotubes, and also into the potential energy required for the deformation of the nanotubes, as shown in Figure 1a and Video S2. For case P, the final kinetic energy is slightly higher than the initial kinetic energy, while the final potential energy is slightly lower than the initial potential energy, indicating that the system is more stable after collision. Furthermore, it can be observed that the highest potential energy (see the marker a in Figure 3b) of case P is higher than those (markers b and c) of cases B and C, which means that the system for case P could have enough collision energy to overcome the fusion barrier of forming a BCN nanotube with a larger diameter, while it is difficult for cases B and C to fuse to form a nanotube.

### 3.2. Electronic Structures

**Figure 4:**
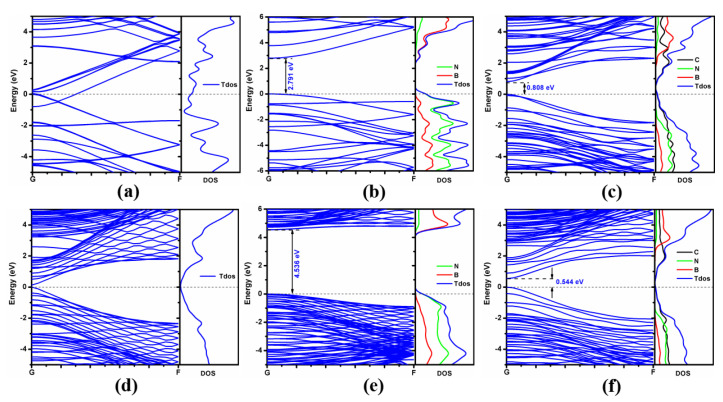
Energy bands and density of states of (6,0) single-walled CNT (**a**), (6,0) single-walled BNNT (**b**), (12,0) single-walled BCN heteronanotube (**c**), GNR (**d**), BNNR (**e**) and BCN heteronanoribbon (**f**).

Figure 4 shows the band structures and density of states (DOS) of the (6,0) single-walled CNT (SWCNT), (6,0) single-walled BNNT (SWBNNT), (12,0) single-walled BCN nanotube (SWBCNNT), graphene nanoribbon (GNR), *h*-BN nanoribbon (BNNR) and BCN heteronanoribbon. It can be seen that the energy levels of (6,0) SWCNT cross the Fermi level (see Figure 4a), which agrees with the consensus that the (6,0) SWCNT is a semimetal [42]. The band gap of (6,0) SWBNNT is calculated to be 2.791 eV (see Figure 4b), in good agreement with the results calculated by Rubio et al. [43,44]. The valence band maximum (VBM) of the (6,0) single-walled BN nanotube is mainly contributed to by N atoms, while the conduction band minimum (CBM) is mainly composed of B atoms (See Figure 4b). The (12,0) single-walled BCN nanotube is a direct band-gap semiconductor with a band gap of 0.808 eV. The VBM is mainly contributed to by C and N atoms, and the CBM is mainly contributed to by C and B atoms (see Figure 4c). The band gap of GNR is close to 0 eV, which is consistent with the results reported by Zhou et al. [45,46]. The band gap of BNNR is calculated to be 4.536 eV, as shown in Figure 4e, which is in good agreement with the results reported by Leite et al. [47]. The VBM of BNNR is mainly composed of N atoms, while the CBM is mainly composed of B atoms. The BCN heteronanoribbon is a direct band-gap semiconductor with a band gap of 0.544 eV (see Figure 4f). The VBM of the BCN heteronanoribbon is composed of C and N atoms, and the CBM is mainly contributed to by C atoms, indicating that C atoms are the main factor that constitutes the small band gap of the BCN heteronanoribbon.

The above calculation results indicate that the electronic properties of CNT/BNNT can be regulated effectively by the collision fusion of CNT and BNNT. In particular, the heteronanotubes and heteronanoribbons formed are new types of semiconductor nanomaterials, which may be applied in electronic devices, photocatalysis, integrated circuits and field effect transistors.

### 3.3. Defects

In these fusion structures attained, some of them contain defects, including vacancy, void, 5–7 pair defect, SW defect, ISW defect and 4–8 ring defect, which can have significant effects on the electrical, chemical and mechanical properties of the fusion structures. It was found that the formation of the 5–7 pair defect is conducive to the fusion of carbon nanotubes [48,49]. Lambin et al. [48] found that the 5–7 pair defects enable the two carbon nanotubes with different helicities to join and form molecular junctions, which had electrical properties different from the original nanotubes. Terrones et al. [49] found that if both carbon nanotubes with the same helicity contain a 5–7 pair defect, a CNT with a larger diameter can be formed through the fusion of the two nanotubes.

**Figure 5:**
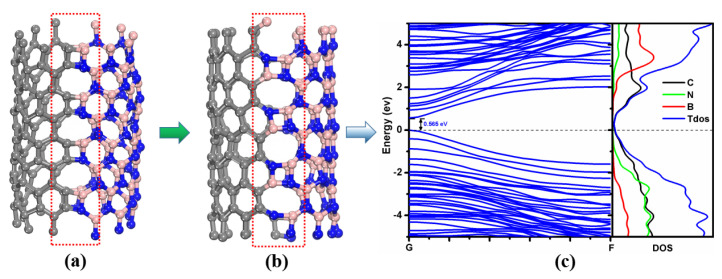
Schematic diagram of BCN heteronanotubes with alternating connections of four- and eight-membered rings. (**a**) Four-membered rings with C-C, C-B, B-N and N-C bonds. (**b**) Four-membered rings with each bond being a B-N type. (**c**) Energy band and density of states corresponding to the structure in (**b**).

Among these defects, the 4–8 ring defect is the most easily formed defect structure after a CNT colliding with a BNNT. During the formation processes of BCN heteronanotube with 4–8 ring defects, four-membered rings containing C-C, C-N, C-B and B-N bonds are formed first, but then they all evolve into four-membered rings containing B-N bonds, as shown in Figure 5a,b. Based on our first-principles calculations, it is found that the energy of the BCN nanotube with B-N-B-N structure is lower than that with C-C-B-N structure. Therefore, it is easy to form four-membered ring structures with all B-N bonds in the evolution process. Figure 5c shows the electronic structure of the BCN nanotube with 4–8 ring defects. As can be seen, the heterostructure is a direct band-gap semiconductor with a band gap of 0.565 eV, which is slightly lower than that of the defect-free BCN heteronanotube. This indicates that the formation of the defects could effectively regulate the properties of the heteronanotube.

In addition, some experimental techniques may be used to reduce the defects in the fused nanostructures. For example, defects can be eliminated by a combination of quenching and annealing methods. Previous SCC-DFTB simulations on the quenching and annealing processes of CNTs and GNRs with defects under a high temperature environment [50] indeed showed that the defects in both CNTs and GNRs could be substantially reduced. Therefore, a combination of quenching and annealing methods can be an effective approach to eliminate defects or reduce the defect density in heterostructures obtained by collision fusion.

## 4. Computational Methods

Collision dynamics simulations were implemented with the DFTB^+^ open source package [51,52,53]. Both CNTs and BNNTs were single-walled nanotubes with a chirality index of (6,0) and a length of about 17.62 Å, containing 192 atoms in total. The initial collision energy (*E*_k_) ranged from 0.1 eV/atom to 1.0 eV/atom with an energy interval of 0.1 eV/atom (the collision energy was calculated from carbon atoms to ensure that the initial collision velocities of the two tubes are the same, and the corresponding collision velocity was varied from 12.67 Å/ps to 40.07 Å/ps). The atomic motion followed through Newton’s equation, which was solved using the velocity Verlet algorithm [54]. The timestep was set to be 1 fs to ensure the conservations of momentum and energy. The total evolution time was 10 ps. The same collision results could be obtained after testing a longer simulation time. The initial temperature of the system was set at 300 K to investigate the collision fusion of CNTs and BNNTs at room temperature. In addition, it is noted that the initial structures of both nanotubes have been first optimized and then relaxed at 300 K, which corresponds to the initial setup of atomic velocities. The initial atomic velocities are very small and have no effect on the collision velocity. Molecular dynamics simulations were implemented in the microcanonical NVE ensemble [55].

**Figure 6:**
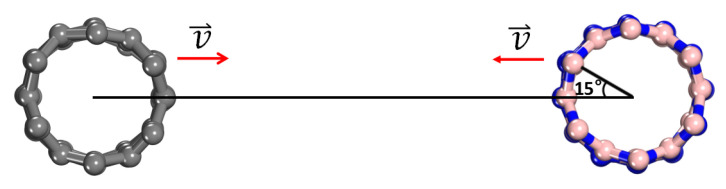
(Color online.) Schematic diagram of the collision setup for (6,0) single-walled CNT (Left) and (6,0) single-walled BNNT (Right) with the same initial velocity but in opposite directions. Gray, blue and pink balls represent carbon, nitrogen and boron atoms, respectively.

**Figure 7:**
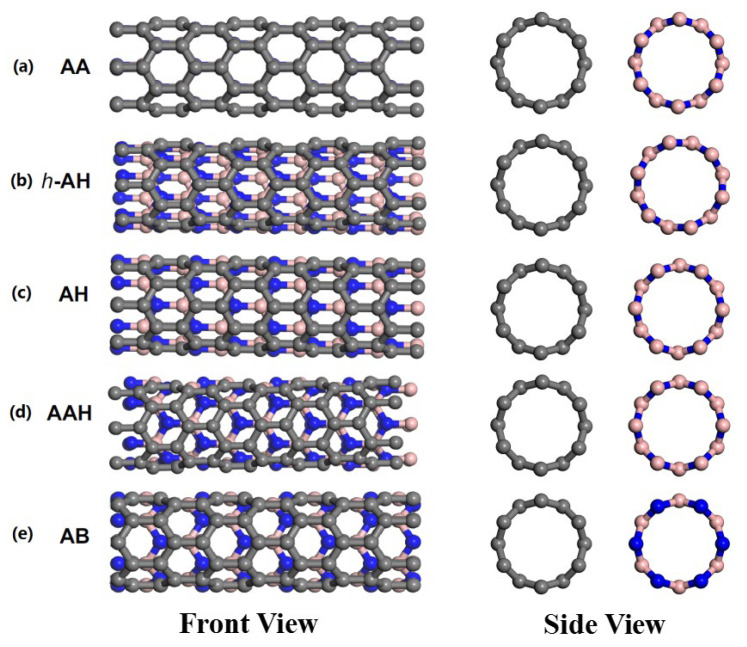
(Color online.) Five collision modes. (**a**) AA, (**b**) *h*-AH, (**c**) AH, (**d**) AAH and (**e**) AB.

All calculations in this paper focused on the parallel collisions of nanotubes, as shown in Figure 6. Considering the symmetry of nanotubes, five collision modes were considered to investigate the effects of collision locations on the collision results. As shown in Figure 7, a string of atoms along the axis in the CNT collided rightly with a string of atoms in the BNNT, the atoms and the centers of hexagonal rings, as well as the centers of bonds, i.e., atoms-colliding-atoms, atoms-colliding-atoms-hexagons, atoms-colliding-bonds, which were denoted as AA, AAH and AB, respectively. On the basis of type AA, CNT remained unchanged, and BN nanotubes were rotated by 15° and 30°, which were called *h*-AH and AH, respectively. In addition, five collision simulations were carried out for each of the five collision modes, and the collision results were statistically averaged for all collision simulations.

In order to ensure that there was no interaction between the two nanotubes at the beginning of the simulations, the initial distance between the walls of two nanotubes was set to be at least 20.00 Å. A periodic boundary condition along the tube axis was applied to simulate a nanotube with infinite length. Considering the presence of boron, carbon and nitrogen atoms in the system, the self-consistent-charge method was performed to avoid undesired electronic states of zwitterions [56].

The first-principles software package CASTEP was used to optimize the fused structure and calculate the electronic structure [57]. The generalized gradient approximation (GGA) parameterized by Perdew, Burke and Ernzerhof (PBE) was applied for the exchange and correlation interactions [58]. The cut-off energy for the plane-wave expansion was set to be 500 eV and the *k*-point was chosen as 0.01 Å^−1^. The force on each ion was less than 0.01 eV/Å and the energy was converged within 1.0 × 10^−5^ eV/atom.

## 5. Conclusions

In this work, the collision dynamics between CNTs and BNNTs under five parallel collision modes of AA, *h*-AH, AH, AAH and AB were investigated by the self-consistent-charge density-functional tight-binding molecular dynamics method. The five main collision outcomes are: the two nanotubes can (1) bounce back, (2) collide into heterojunctions, (3) fuse into BCN heteronanotubes, (4) form BCN heteronanoribbons and (5) collide to cause serious damage after collision. There may be defects created in the fused structure, including vacancy, void and irregular defects such as the 5–7 pair defect, SW defect, ISW defect and 4–8 ring defects. With the increase in collision energy, the number of defects increases, and the nanotubes may even break up into single atoms, dimers, trimers or chains of atoms. In addition, the electronic structures of BCN heteronanotubes and BCN heteronanoribbons formed by collision were investigated based on first-principles calculations. It was found that both the defect-free (12,0) BCN heteronanotube and the BCN heteronanoribbon are direct band-gap semiconductors with band gaps of 0.808 eV and 1.34 eV, respectively. These results indicate that the electronic structures of nanotubes can be effectively tuned by collision of CNTs and BNNTs, which could have significant implications in the field of electronic devices, photocatalysis, integrated circuits and field effect transistors. Although the present work only focuses on the collision fusion of CNTs and BNNTs with specific helicity, it can be extended to the collision processes of nanotubes with any helicity. Therefore, the present work not only theoretically demonstrates a novel method to create a heterostructure via collision fusion but also gains in-depth understanding in the synthesis of heteronanotubes and heteronanoribbons that can guide experiments.

## Figures and Tables

**Table 1:** Results of collision between SWCNT and SWBNNT. Table legend: B = bounce back, C = connecting heterojunction, P = perfect BCN nanotube with no defects, D = BCN nanotube with defects, S = serious damage, En = Energy.

		Model	AA	*h*-AH	AH	AAH	AB
	Result						
En (eV)							
0.1			B, C, P	B, C, P	B, C	B	B, C
0.2			B, C	B, C, P	B, P	B	B, C, P
0.3			B, C	B, P, D	B, P, D	B, C, P, D	B, C, P
0.4			B, P, D	P, D	B, D	D	B, P, D
0.5			D	D, S	B, P, D	B, D, S	P, D, S
0.6			D, S	D, S	P, D, S	D, S	P, D, S
0.7			S	S	B, D, S	S	P, D, S
0.8			S	S	S	D, S	S
0.9			S	S	D, S	D, S	S
1.0			S	S	S	S	S

## Data Availability

The data that support the findings of this study are available from the corresponding author upon reasonable request.

## Supplementary Materials

The following supporting information can be downloaded at https://www.mdpi.com/article/10.3390/molecules28114334/s1, Video S1: Bouncing off of two nanotubes after collision. Video S2: Merging together of two nanotubes after collision. Video S3: Forming BCN nanotube with no defect. Video S4: Forming BCN nanotube with defects.

## References

[1] Wu H., Wang Y.J., Xu Y.F., Sivakumar P.K., Pasco K., Filippozzi U., Parkin S.S.P., Zeng Y.J., McQueen T., Ali M.N. (2022). The field-free Josephson diode in a van der Waals heterostructure. Nature.

[2] Ren W.J., Ouyang Y.L., Jiang P.F., Yu C.Q., He J., Chen J. (2021). The Impact of Interlayer Rotation on Thermal Transport Across Graphene/Hexagonal Boron Nitride van der Waals Heterostructure. Nano Lett..

[3] Li Y., Zhang J.W., Chen Q.G., Xia X.H., Chen M.H. (2021). Emerging of Heterostructure Materials in Energy Storage: A Review. Adv. Mater..

[4] Huang S.Z., Wang Z.H., Lim Y.V., Wang Y., Li Y., Zhang D.H., Yang H.Y. (2021). Recent Advances in Heterostructure Engineering for Lithium–Sulfur Batteries. Adv. Energy Mater..

[5] Tang F.H., He D.X., Jiang H., Wang R.S., Li Z.L., Xue W.D., Zhao R. (2022). The coplanar graphene oxide/graphite heterostructure-based electrodes for electrochemical supercapacitors. Carbon.

[6] Yuan K., Hao P.J., Zhou Y., Hu X.C., Zhang J.B., Zhong S.W. (2022). A two-dimensional MXene/BN van der Waals heterostructure as an anode material for lithium-ion batteries. Phys. Chem. Chem. Phys..

[7] Liu C., Lu Y.H., Yu X.T., Shen R.J., Wu Z.M., Yang Z.S., Yan Y.F., Feng L.X., Lin S.S. (2022). Hot carriers assisted mixed-dimensional graphene/MoS_2_/p-GaN light emitting diode. Carbon.

[8] Wang B.A., Yuan H.K., Yang T., Wang P., Xu X.H., Chang J.L., Kuang M.Q., Chen H. (2022). A two-dimensional PtS2/BN heterostructure as an S-scheme photocatalyst with enhanced activity for overall water splitting. Phys. Chem. Chem. Phys..

[9] Song J., Jiang M.J., Wan C., Li H.J., Zhang Q., Chen Y.H., Wu X.H., Yin X.M., Liu J.F. (2022). Defective graphene/SiGe heterostructures as anodes of Li-ion batteries: A first-principles calculation study. Phys. Chem. Chem. Phys..

[10] Sun X.X., Zhu C.G., Yi J.L., Xiang L., Ma C., Liu H.W., Zheng B.Y., Liu Y., You W.X., Zhang W.J. (2022). Reconfigurable logic-in-memory architectures based on a two-dimensional van der Waals heterostructure device. Nat. Electron..

[11] Zheng Q., Zhuang Y.C., Sun Q.F., He L. (2022). Coexistence of electron whispering-gallery modes and atomic collapse states in graphene/WSe2 heterostructure quantum dots. Nat. Commun..

[12] Chepkasov I.V., Smet J.H., Krasheninnikov A.V. (2022). Single- and Multilayers of Alkali Metal Atoms inside Graphene/MoS_2_ Heterostructures: A Systematic First-Principles Study. J. Phys. Chem. C.

[13] Yang Z.H., Wu M.S., Luo W.W., Liu G., Xu B. (2022). Structural, Electronic, and Transport Properties of Phosphorene–Graphene Lateral Heterostructure Anodes: Insights from First-Principles Calculations. J. Phys. Chem. C.

[14] Kuang H.F., Zhang H.Q., Liu X.H., Chen Y.D., Zhang W.G., Chen H., Ling Q.D. (2022). Microwave-assisted synthesis of NiCo-LDH/graphene nanoscrolls composite for supercapacitor. Carbon.

[15] Wang H., Chen J.M., Lin Y.P., Wang X.H., Li J.M., Li Y., Gao L.J., Zhang L.B., Chao D.L., Xiao X. (2021). Electronic Modulation of Non-van der Waals 2D Electrocatalysts for Efficient Energy Conversion. Adv. Mater..

[16] Song S., Gong J., Jiang X.W., Yang S.Y. (2022). Influence of the interface structure and strain on the rectification performance of lateral MoS_2_/graphene heterostructure devices. Phys. Chem. Chem. Phys..

[17] Iijima S. (1991). Helical microtubules of graphitic carbon. Nature.

[18] Han L.Y., Xiao C.X., Song Q., Yin X.M., Li W., Li K.Z., Li Y.Y. (2022). Nano-interface effect of graphene on carbon nanotube reinforced carbon/carbon composites. Carbon.

[19] Zhang S., Pang J.B., Li Y.F., Yang F., Gemming T., Wang K., Wang X., Peng S.G., Liu X.Y., Chang B. (2022). Emerging Internet of Things driven carbon nanotubes-based devices. Nano Res..

[20] Wang R.R., Wu R.B., Yan X.X., Liu D., Guo P.F., Li W., Pan H.G. (2022). Implanting Single Zn Atoms Coupled with Metallic Co Nanoparticles into Porous Carbon Nanosheets Grafted with Carbon Nanotubes for High-Performance Lithium-Sulfur Batteries. Adv. Funct. Mater..

[21] Zhu R.F., Wang D., Liu Y.M., Liu M.M., Fu S.H. (2022). Bifunctional superwetting carbon nanotubes/cellulose composite membrane for solar desalination and oily seawater purification. Chem. Eng. J..

[22] Poggioli A.R., Limmer D.T. (2021). Distinct Chemistries Explain Decoupling of Slip and Wettability in Atomically Smooth Aqueous Interfaces. J. Phys. Chem. Lett..

[23] Stern H.L., Gu Q.S., Jarman J., Barker S.E., Mendelson N., Chugh D., Schott S., Tan H.H., Sirringhaus H., Aharonovich I. (2022). Room-temperature optically detected magnetic resonance of single defects in hexagonal boron nitride. Nat. Commun..

[24] Pham P.V., Bodepudi S.C., Shehzad K., Liu Y., Xu Y., Yu B., Duan X.F. (2022). 2D Heterostructures for Ubiquitous Electronics and Optoelectronics: Principles, Opportunities, and Challenges. Chem. Rev..

[25] Turiansky M.E., Alkauskas A., van de Walle C.G. (2020). Spinning up quantum defects in 2D materials. Nat. Mater..

[26] Mirzayev M.N. (2021). Heat transfer of hexagonal boron nitride (h-BN) compound up to 1 MeV neutron energy: Kinetics of the release of wigner energy. Radiat. Phys. Chem..

[27] Xu T., Zhang K., Cai Q.R., Wang N.Y., Wu L.Y., He Q., Wang H., Zhang Y., Xie Y.F., Yao Y.G. (2022). Advances in synthesis and applications of boron nitride nanotubes: A review. Chem. Eng. J..

[28] Qi R.S., Li N., Du J.L., Shi R.C., Huang Y., Yang X.X., Liu L., Xu Z., Dai Q., Yu D.P. (2021). Four-dimensional vibrational spectroscopy for nanoscale mapping of phonon dispersion in BN nanotubes. Nat. Commun..

[29] Konabe S. (2021). Exciton effect on shift current in single-walled boron-nitride nanotubes. Phys. Rev. B.

[30] Feng Y., Li H.N., Hou B., Kataura H., Inoue T., Chiashi S., Xiang R., Maruyama S. (2021). Zeolite-supported synthesis, solution dispersion, and optical characterizations of single-walled carbon nanotubes wrapped by boron nitride nanotubes. J. Appl. Phys..

[31] Jones R.S., Maciejewska B., Grobert N. (2020). Synthesis, characterisation and applications of core–shell carbon–hexagonal boron nitride nanotubes. Nanoscale Adv..

[32] Stephan O., Ajayan P.M., Colliex C., Redlich P., Lambert J.M., Bernier P., Lefin P. (1994). Doping Graphitic and Carbon Nanotube Structures with Boron and Nitrogen. Science.

[33] Wang Y., Huang G., Zhang J., Shao Q.Y. (2014). Tunable electronic properties of ultra-thin boron-carbon-nitrogen heteronanotubes for various compositions. J. Mol. Model..

[34] Chaudhuri P., Lima C.N., Frota H.O., Ghosh A. (2019). First-principles study of nanotubes of carbon, boron and nitrogen. Appl. Surf. Sci..

[35] Belgacem A.B., Hinkov I., Yahia S.B., Brinza O., Farhat S. (2016). Arc discharge boron nitrogen doping of carbon nanotubes. Mater. Today Commun..

[36] Campbell E.E.B., Schyja V., Ehlich R., Hertel I.V. (1993). Observation of molecular fusion and deep inelastic scattering in C_60_^2+^ + C_60_ collisions. Phys. Rev. Lett..

[37] Rohmund F., Campbell E.E.B. (1995). Charge transfer collisions between C_60_^2+^ and C_60_. Chem. Phys. Lett..

[38] Rohmund F., Glotov A., Hansen K., Campbell E.E.B. (1996). Experimental studies of fusion and fragmentation of fullerenes. J. Phys. B.

[39] Lee G.D., Wang C.Z., Yoon E., Hwang N.M., Ho K.M. (2010). The role of pentagon–heptagon pair defect in carbon nanotube: The center of vacancy reconstruction. Appl. Phys. Lett..

[40] Stone A.J., Wales D.J. (1986). Theoretical studies of icosahedral C_60_ and some related species. Chem. Phys. Lett..

[41] Lusk M.T., Wu D.T., Carr L.D. (2010). Graphene nanoengineering and the inverse Stone-Thrower-Wales defect. Phys. Rev. B.

[42] Odom T.W., Huang J.L., Kim P., Lieb C.M. (1998). Atomic structure and electronic properties of single-walled carbon nanotubes. Nature.

[43] Rubio A., Corkill J.L., Cohen M.L. (1994). Theory of graphitic boron nitride nanotubes. Phys. Rev. B.

[44] Blasé X., Rubio A., Louie S.G., Cohen M.L. (1994). Stability and Band Gap Constancy of Boron Nitride Nanotubes. Europhys. Lett..

[45] Zhou S.Y., Gweon G.H., Fedorov A.V., First P.N., De Heer W.A., Lee D.H., Guinea F., Castro Neto A.H., Lanzara A. (2007). Substrate-induced bandgap opening in epitaxial graphene. Nat. Mater..

[46] Son Y.W., Cohen M.L., Louie S.G. (2006). Energy Gaps in Graphene Nanoribbons. Phys. Rev. Lett..

[47] Zhang Z.H., Guo W.L. (2008). Energy-gap modulation of BN ribbons by transverse electric fields: First-principles calculations. Phys. Rev. B.

[48] Lambin P., Vigneron J.P., Fonseca A., Nagy J.B., Lucas A.A. (1996). Atomic structure and electronic properties of a bent carbon nanotube. Synth. Met..

[49] Terrones M., Terrones H., Banhart F., Charlier J.-C., Ajayan P.M. (2000). Coalescence of Single-Walled Carbon Nanotubes. Science.

[50] Zhang C., Mao F., Meng X.R., Wang D.Q., Zhang F.S. (2016). Collision-induced fusion of two single-walled carbon nanotubes: A quantitative study. Chem. Phys. Lett..

[51] Aradi B., Hourahine B., Frauenheim T. (2007). DFTB+, a Sparse Matrix-Based Implementation of the DFTB Method. J. Phys. Chem. A.

[52] Enyashin A.N., Ivanovskii A.L. (2005). Mechanical and electronic properties of a C/BN nanocable under tensile deformation. Nanotechnology.

[53] Enyashin A.N., Seifert G., Ivanovskii A.L. (2005). Calculation of the Electronic and Thermal Properties of C/BN Nanotubular Heterostructures. Inorg. Mater..

[54] Swope W.C., Andersen H.C., Berens P.H., Wilson K.R. (1982). A computer simulation method for the calculation of equilibrium constants for the formation of physical clusters of molecules: Application to small water clusters. J. Chem. Phys..

[55] Kraska T. (2006). Molecular-dynamics simulation of argon nucleation from supersaturated vapor in the NVE ensemble. J. Chem. Phys..

[56] Jakowski J., Irle S., Morokuma K. (2010). Collision-induced fusion of two C_60_ fullerenes: Quantum chemical molecular dynamics simulations. Phys. Rev. B.

[57] Segall M.D., Lindan P.J.D., Probert M.J., Pickard C.J., Hasnip P.J., Clark S.J., Payne M.C. (2002). First-principles simulation: Ideas, illustrations and the CASTEP code. J. Phys. Condens. Matter..

[58] Perdew J.P., Burke K., Ernzerhof M. (1996). Generalized Gradient Approximation Made Simple. Phys. Rev. Lett..

